# Atomistic simulations on the carbidisation processes in Pd nanoparticles†

**DOI:** 10.1039/d2ra07462a

**Published:** 2023-02-14

**Authors:** Apostolos Kordatos, Khaled Mohammed, Reza Vakili, Alexandre Goguet, Haresh Manyar, Emma Gibson, Marina Carravetta, Peter Wells, Chris - Kriton Skylaris

**Affiliations:** a School of Chemistry, University of Southampton SO17 1BJ UK C.Skylaris@soton.ac.uk; b School of Chemistry and Chemical Engineering Queen's University Belfast BT7 1NN UK; c School of Chemistry, University of Glasgow G12 8QQ UK

## Abstract

The formation of interstitial PdC_*x*_ nanoparticles (NPs) is investigated through DFT calculations. Insights on the mechanisms of carbidisation are obtained whilst the material's behaviour under conditions of increasing C-concentration is examined. Incorporation of C atoms in the Pd octahedral interstitial sites is occurring through the [111] facet with an activation energy barrier of 19.3–35.7 kJ mol^−1^ whilst migration through the [100] facet corresponds to higher activation energy barriers of 124.5–127.4 kJ mol^−1^. Furthermore, interstitial-type diffusion shows that C will preferentially migrate and reside at the octahedral interstitial sites in the subsurface region with limited mobility towards the core of the NP. For low C-concentrations, migration from the surface into the interstitial sites of the NPs is thermodynamically favored, resulting in the formation of interstitial carbide. Carbidisation reaction energies are exothermic up to 11–14% of C-concentration and slightly vary depending on the shape of the structure. The reaction mechanisms turn to endothermic for higher concentration levels showing that C will preferentially reside on the surface making the interstitial carbide formation unfavorable. As experimentally observed, our simulations confirm that there is a maximum concentration of C in Pd carbide NPs opening the way for further computational investigations on the activity of Pd carbides in directed catalysis.

## Abstract 2

The carbidisation mechanisms in PdC_*x*_ nanoparticles are investigated through DFT. Insights on the preferential migration pathways from the surface to the interior are obtained towards the maximum interstitial C concentration.

## Graphical abstract

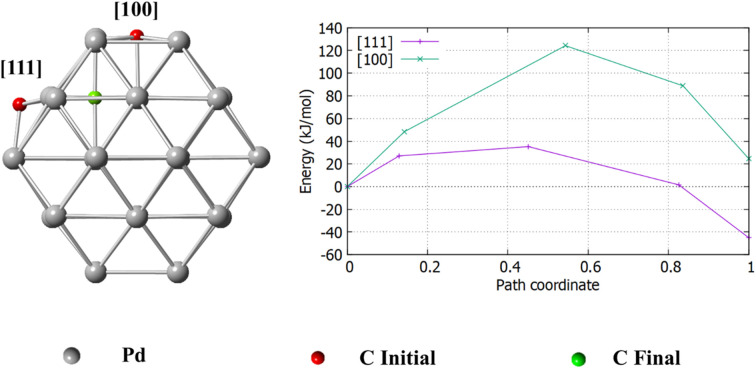

## 1. Introduction

Within heterogeneous catalysis, supported Pd nanoparticles (NPs) have been extensively investigated^1–7^ for their high activity towards many existing and emerging industrial applications;^8,9^ from the selective hydrogenation and oxidation of unsaturated hydrocarbons, to the conversion of biomass,^10,11^ and exhaust emission control technologies. One key example is the semi-hydrogenation of acetylene, which is a crucial purification step for ethylene feedstocks used in polymerization to produce polyethylene (∼50 megatons per year). Elsewhere, as the chemical industry pivots towards more sustainable feedstocks, Pd has proved very effective for the selective upgrading of waste biomass. In these example processes, the Pd structures are significantly altered by the catalytic conditions, forming interstitial carbide structures.

Whether Pd forms interstitial structures of hydride, carbide, or nitride during catalysis, these changes are not innocent as they have a fundamental contribution to the catalytic process. For instance, in acetylene semi-hydrogenation, the formation of hydride phases (α-PdH_*x*_ and β-PdH_*x*_)^12–14^ is considered a rate-limiting factor as full hydrogenation to ethane occurs through reversed hydrogen diffusion on the Pd surface. Carbidic Pd forms *via* incorporation of C atoms into the Pd lattice, suppressing the formation of hydride phases and thus increasing selectivity towards production of ethylene. Similarly, for the hydrogenation of furfural, PdC_*x*_ affects the adsorption of species whilst reducing catalytic turnover. Understanding the formation and stability of carbidic Pd structures is crucial to determine their function in catalysis. Detailed information on the material's controlled synthesis (interactions on the surface and subsurface regions, interstitial C distribution) as well as insights on its behaviour in reaction conditions (deactivation) are still required. Since the adsorption type and microkinetics of hydrocarbons on these structures may vary, atomistic simulations through Density Functional Theory (DFT) and microkinetic modelling can provide advanced insights and complement with experimental methods such as X-ray absorption fine structure (XAFS) on the mechanistic investigation of carbidisation processes and reaction pathways of catalytic mechanisms.

In the study by Usoltsev *et al.*,^15^ the adsorption of ethylene (C_2_H_4_) on the Pd surface is followed by dehydrogenation to C_2_H_3_, C_2_H_2_ and C_2_H leading to the formation of the carbide phase. Experimental results also show the connection of the carbide formation with the Pd lattice expansion^5,16^ whilst Liu *et al.*^17^ highlights the improved catalytic behaviour of PdC_*x*_ through the formation of more reactive sites. The influence of the support in the surface/subsurface chemistry has been also recently highlighted by Rötzer *et al.*^18^ showing different turnover frequencies for two amorphous SiO_2_ films. The catalytic activity between pristine Pd clusters and surface models have been also compared through experimental and theoretical methods on Pd_30_/Al_2_O_3_.^3^ Notably, for the carbidic Pd, the exact amount of C incorporation into the Pd lattice is still debated. Reported values of C-concentration on the PdC_*x*_ do vary from 0.13 to 0.18.^19,20^ Several models for the C dissolution have been proposed in theoretical studies from ordered to completely random distribution in the Pd lattice. Seriani *et al.*^21^ have reported the formation of a metastable carbide that corresponds to 13–14% of C concentration in which hydrogen diffusion is hindered as compared to pristine Pd. This Pd_6_C metastable carbide formation has been further investigated through experimental^22^ and theoretical methods.^23^ In the recent study of He *et al.*, C diffusion into the Pd(100) and Pd(111) surface models show zigzag trajectories for the migration pathways and a non-uniform distribution in the slab. Furthermore, exothermic behaviour for C migration is observed through the Pd(111) facet and endothermic for Pd(100) whilst the stability decreases with the insertion depth. Schuster *et al.* propose that the formation kinetics depend on the particle size whilst shape also influences hydrogen assisted reactions.^24^ The selectivity of the catalyst has also been reported to be affected by the particle size.^25,26^ Torres *et al.*^27^ have also examined the impact of different experimental conditions, showing that the dissolution of C is affected by the application of tensile stress on the surface. Additionally, the adsorption and conversion of hydrocarbons are affected by the surface morphology^28^ (flat *vs.* stepped), whilst similarly the expansion of Pd lattice on NPs close to the surface should be also considered since the formation of subsurface Pd carbides is expected to be hindered by the neighboring C–C repulsion. C incorporation in the Pd subsurface affects the adsorption type of species making the catalyst selective towards specific intermediates and products.^29^ It is therefore evident that the actual role of the carbide in selective hydrogenation depends on the formation process and distribution of C atoms into the pristine Pd. To this point, the aforementioned works have mostly focused on Pd bulk or surface/slab crystallographic arrangements, however, the formation mechanism in Pd NPs has not been reported.

In this study, we perform DFT calculations to investigate the PdC_*x*_ formation on Pd nanoparticles of different crystallographic arrangements. Firstly, C surface residing and migration into the subsurface Pd octahedral interstitial sites is examined whilst the distribution of the dopant in the Pd host lattice is also considered through the interstitial-type diffusion *via* neighboring positions. Additional monitoring on the PdC_*x*_ formation at increasing C-concentration is also considered to estimate the maximum amount for each structure. The aim of this work is to better understand the formation of Pd carbides and their role in catalysis.

## 2. Experimental

The pristine Pd and PdC_*x*_ NPs structures are modelled through the linear – scaling DFT code ONETEP.^30^ The density matrix is constructed by using localized nonorthogonal Wannier functions (NGWFs) as expressed through a set of periodic sinc (p-sinc) functions.^31^ NGWFs are equivalent to plane waves for more conventional DFT codes and for our calculations the p-sinc basis set was set to a kinetic energy cut-off of 800 eV whilst for the exchange and correlation interactions, the formulation with the corrected density functional of Perdew, Burke and Ernzerhof (PBE)^32^ within the generalized gradient approximation (GGA) is applied with norm – conserving pseudopotentials. The density matrix and NGWFs are optimized *via* two separate loops. Since this work focuses on a metallic system, the Ensemble DFT (EDFT) method^33^ is employed with a Fermi-Dirac smearing of 0.1 eV under constant pressure conditions allowing the cells to relax in the minimum energy configuration. An NGWF radius of 9.0 Bohr has been used for all structures, whilst geometry optimization is performed at the Γ-point in cells of 18–22 Å.

For the construction of NPs crystallographic arrangements, the ASE^34^ tool has been used with the average Pd–Pd bond for all optimized structures corresponding to an average of 2.74 Å. After determining the relative energies of reactants and products, the relaxed configurations of Pd NPs are then used as input for the transition states investigation along a minimum energy reaction path. For the estimation of the activation energy barriers, reaction energies and location of the transition state the Linear Synchronous Transit/Quadratic Synchronous Transit (LSTQST)^35^ protocol is employed. Initially, a rapid scan along the reaction/transition path is performed followed by a located minimization to find the most stable intermediate. The schematic representation of the Pd and PdC_*x*_ cells is generated using the CrystalMaker^36^ software whilst for the reaction energies between different crystallographic configurations the following formula is used:*E*_reaction_ = *E*_products_ − *E*_reactants_where *E*_products_ corresponds to the energy of the relaxed structures of products and *E*_reactants_ to the energy of the relaxed structures of the reactants.

## 3. Results and discussion

### 3.1 Crystallographic arrangements

**Fig. 1:**
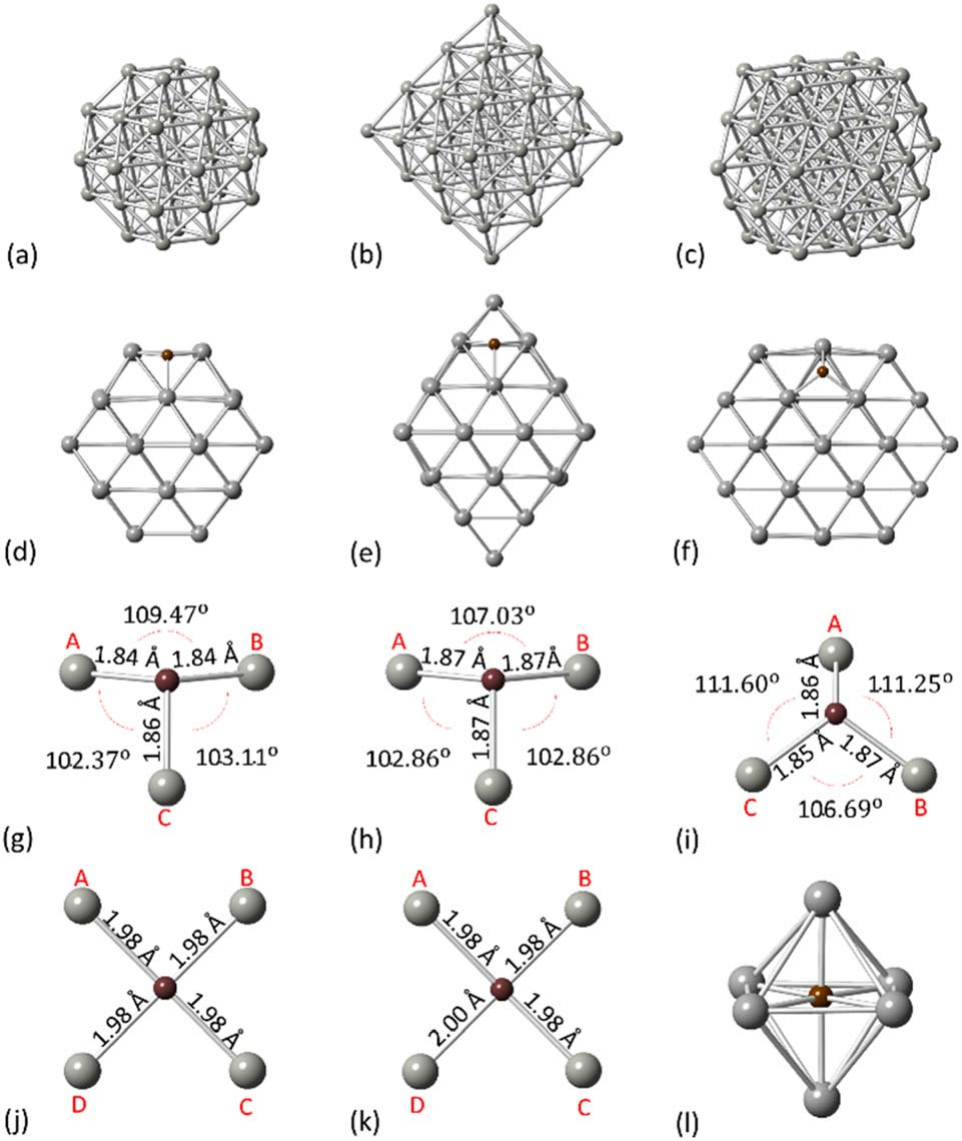
Schematic representation for (a) Pd_38_ truncated octahedron (b) Pd_44_ octahedron (c) Pd_55_ cuboctahedron (d) C residing on the [111] facet of Pd_38_ (e) C residing on the [111] facet of Pd_44_ (f) C residing on the [111] facet of Pd_55_ (g) C bonding on the [111] facet of Pd_38_ (h) C bonding on the [111] facet of Pd_44_ (i) C bonding on the [111] facet of Pd_55_ (j) C bonding on the [100] facet of Pd_38_ (k) C bonding on the [100] facet of Pd_55_ and (l) C in the Pd octahedral site. Pd atoms for grey spheres and C atoms for brown spheres.

Initially, we considered the NPs crystallographic arrangements to explore the carbidisation process on different facets. For our calculations, the PdC_*x*_ NPs are modelled using truncated octahedral Pd_38_, octahedral Pd_44_ and cuboctahedral Pd_55_ (Fig. 1a–c). The geometries have been considered as appropriate to represent a range of nanoparticle structures from both experimental and computational approaches. Additionally, we aim to account for the effect of different facets on the carbidisation mechanisms through their coexistence on the same particle whilst, a comparison of outcomes for a range of different structures was required to validate results.

All NPs have been modelled as isolated structures in conditions of vacuum to avoid interactions with their periodic images. Initially, we assessed how C resides on the surface of Pd NPs. Therefore, we investigated the preferential configurations of the surface Pd–C interactions as an initial process from C exposure, prior to the interstitial carbide formation. Fig. 1d–f represents the preferential configurations for C on Pd_38_, Pd_44_ and Pd_55_ on the [111] facet. For all structures, an individual C atom will bind with three surface Pd atoms (hollow site) with the average C–Pd bond length being 1.85 Å, 1.87 Å and 1.86 Å for Pd_38_, Pd_44_ and Pd_55_, respectively (Fig. 1g–i). In addition, C binds with four surface Pd atoms on the [100] facet as shown in Fig. 1j and k whilst the average C–Pd bond length is 1.98 Å and 1.99 Å for Pd_38_ and Pd_55_, respectively. For the interstitial formation, each C atom is in the middle of the Pd octahedral site which is the minimum energy configuration,^37^ binding with six Pd atoms (Fig. 1l). The structural changes of the Pd NPs surface due to binding with an individual C are summarized in Table 1.

Structural deformations of the Pd NP surface after C binding. Surface Pd–Pd bond lengths (AB, BC, CA, CD, DA) as illustrated in Fig. 1g–k, correspond to Pd_38_, Pd_44_ and Pd_55_ for the [111] and [100] facets, respectively

**Table:** 

[111]	Pd–Pd (Å) – pristine			Pd–Pd (Å) – surface C		
Bond	AB	BC	CA	AB	BC	CA
Pd_38_	2.72	2.72	2.74	2.87	2.95	2.92
Pd_44_	2.77	2.69	2.69	2.96	2.91	2.91
Pd_55_	2.75	2.75	2.75	3.02	3.01	3.02

**Table:** 

[100]	Pd–Pd (Å) – pristine				Pd–Pd (Å) – surface C			
Bond	AB	BC	CD	DA	AB	BC	CD	DA
Pd_38_	2.75	2.75	2.75	2.75	2.79	2.79	2.79	2.79
Pd_55_	2.75	2.75	2.75	2.75	2.79	2.79	2.82	2.82

### 3.2 Carbidisation processes

After establishing the initial and final positions of C, we investigated the process of migration from the surface into the interior of the particle *via* transition state search calculations. In previous work, Crespo-Quesada *et al.*^38^ showed on cubic and octahedral NPs, that the carbidisation rate on Pd [111]/octahedral NPs was lower as compared with Pd [100]/cubes and attributed this to the stronger binding of acetylene with the [100] 4-fold sites. In their study, which did not include transition state searches, DFT calculations were performed on Pd_36_C_*n*_ (*n* = 1, 2 or 6). This provided useful insights, however it is limited in terms of different shapes and sizes of NP. In this work, we look at the actual mechanism of carbidisation in NPs of different shapes and sizes, alongside how the extent of C incorporation (∼2–20%) affects the carbidisation process.

**Fig. 2:**
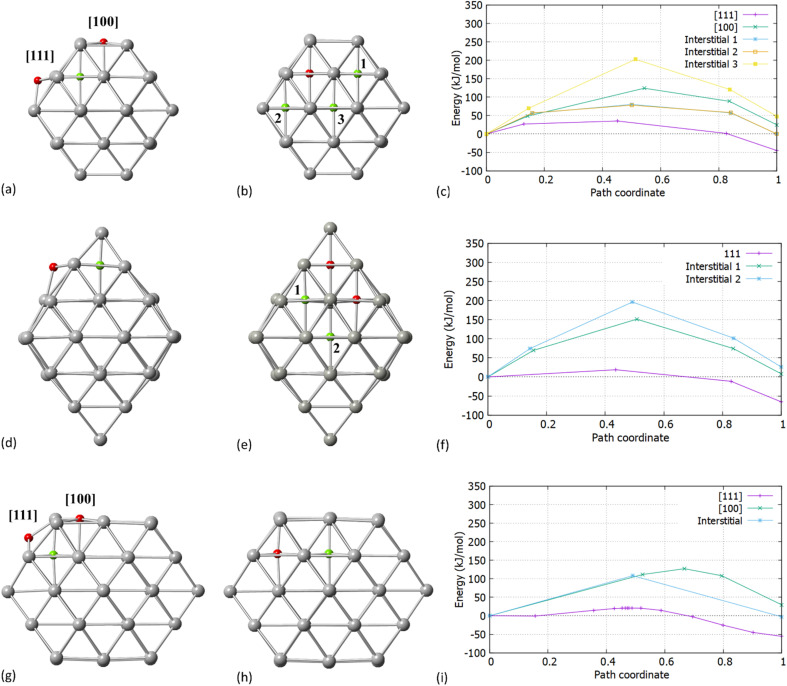
Activation energy barriers for the carbidisation mechanisms in Pd NPs (a) transition states for C migration in Pd_38_ through the [100] and [111] facets (b) interstitial-type diffusion of C for neighboring sites in Pd_38_ (c) activation energies for surface and interstitial-type mobilities in Pd_38_ (d) transition states for C migration in Pd_44_ through the [100] and [111] facets (e) interstitial-type diffusion of C for neighboring sites in Pd_44_ (f) activation energies for surface and interstitial-type mobilities in Pd_44_ (g) transition states for C migration in Pd_55_ through the [100] and [111] facets (h) interstitial-type diffusion of C for neighboring sites in Pd_55_ and (i) activation energies for surface and interstitial-type mobilities in Pd_55_. Red spheres correspond to initial positions of C and green spheres to final positions of C.

Our initial expectation was that C resides initially on the surface of both [100] and [111] facets for Pd_38_/Pd_55_. We found that C will preferentially migrate into the Pd NP interstitial sites through the [111] facet. Fig. 2 present the surface and interstitial diffusion mechanisms for Pd_38_, Pd_44_ and Pd_55,_ respectively as assessed through the transition states investigation. Firstly, for all Pd NPs, the reaction energy for the C migration through the [111] facet is exothermic. The activation energy barriers for C incorporation through the [111] facet correspond to 35.7 kJ mol^−1^, 19.3 kJ mol^−1^ and 21.2 kJ mol^−1^ for Pd_38_, Pd_44_ and Pd_55_, respectively.

When we considered the cuboctahedral Pd_55_ and truncated octahedral Pd_38_ NPs, the insertion reaction from the [100] facet into the particle is endothermic and requires a higher activation energy barrier of 124.5 kJ mol^−1^ and 127.4 kJ mol^−1^ for Pd_38_ and Pd_55_, respectively, making C insertion less energetically favourable. We expect that even if the [100] facet strongly binds with hydrocarbons, it will limit the reaction process and have a minor contribution to the total carbidisation rate. In contrast, the exothermic behavior for [111] as represented in Fig. 2c and i shows that C atoms will migrate into the NP through the [111] facet and reside in the octahedral Pd interstitial sites. Additionally, the calculated reaction energies for the mechanism are −45.05 kJ mol^−1^, −65.45 kJ mol^−1^ and −55.42 kJ mol^−1^ for Pd_38_, Pd_44_ and Pd_55_, respectively. This demonstrates that the process is slightly more favoured for the octahedral Pd_44_. Interestingly, considering the shape difference for Pd_38_, Pd_44_ and Pd_55_ there is a relatively small activation energy barrier for carbidisation in all cases for the [111] facet. The results on the activation energy barriers for C migration are in good agreement (36.6–40.5 kJ mol^−1^) with previously reported values on slab models.^23^ The energies of reaction and relevant barriers considering C incorporation from the surface into the NP for all structures are summarized in Table 2.

Activation energy barriers and reaction energies for the surface to interstitial C migration mechanisms in Pd_38_, Pd_44_ and Pd_55_ through the [111] and [100] facets, respectively

**Table:** 

[111]	*E*_act_ (kJ mol^−1^)	*E*_reaction_ (kJ mol^−1^)
Pd_38_	35.7	−45.05
Pd_44_	19.3	−65.45
Pd_55_	21.2	−55.42

**Table:** 

[100]	*E*_act_ (kJ mol^−1^)	*E*_reaction_ (kJ mol^−1^)
Pd_38_	124.5	24.68
Pd_55_	127.4	29.54

Whilst the first part of our calculations focused on the C migration from the surface to interstitial site, we then considered the C mobility into the NP *via* examining interstitial-type diffusion mechanisms. For Pd_38_, a corresponding energy barrier of 77.2 kJ mol^−1^ was found when migrating through neighboring sites at the subsurface region. Additionally, a considerable increase in the activation energy is observed when migrating towards the core of the particle (193.5 kJ mol^−1^) as shown in Fig. 2c. After C insertion into a subsurface interstitial site, C will preferentially move through nearest positions close to the surface rather than further diffuse into the interior of the Pd lattice. This is an important finding when considering the maximum amount of C that can be incorporated into a given NP; higher C loadings require full occupation of the octahedral interstitial sites, however, there is a strong preference for C atoms to reside in the subsurface area. For octahedral Pd_44_, as shown in Fig. 2e and f a higher activation energy barrier of 151.5 kJ mol^−1^ and 196.8 kJ mol^−1^ is required for C migration towards the subsurface and the core, respectively. With these higher barriers it is expected that C will reside on the initially occupied positions with limited mobility into neighboring sites. The C subsurface diffusion mechanisms, as well as the C–C repulsion forces have been considered in this study as design criteria on pre-formed carbidic structures to investigate the carbidisation mechanisms at increasing C concentrations. A criterion we take into account when we design carbidised nanoparticles is to allow at least an unoccupied interstitial site between neighboring carbon atoms. It is also observed that at higher concentrations, interstitial subsurface C binds with less than 6 Pd atoms, residing towards the surface of the NP.

### 3.3 Carbide formation at higher C-concentration

**Fig. 3:**
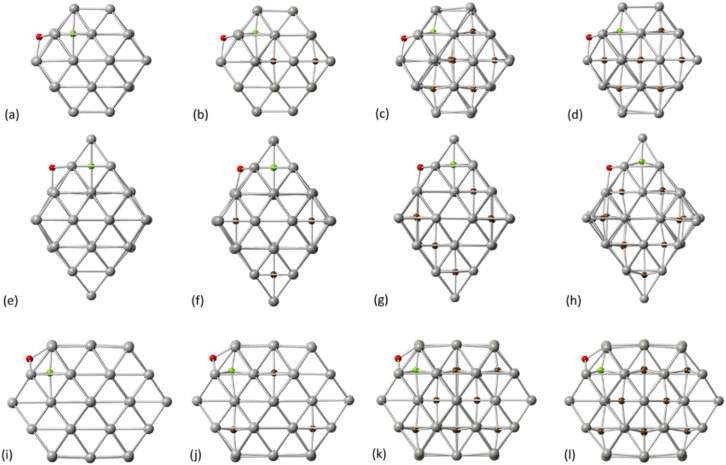
Transition states for the carbidisation mechanisms at increasing C concentration for Pd_38_ – (a) 2.6% (b) 9.5% (c) 15.6% and (d) 20.8%, Pd_44_ – (e) 2.2% (f) 8.3% (g) 15.4% and (h) 21.4%, Pd_55_ – (i) 1.8% (j) 8.3% (k) 15.4% and (l) 20.3%. Red spheres correspond to initial positions of C and green spheres to final positions of C.

To further investigate the experimentally confirmed limitations of C concentration in Pd NPs, we examined the activation energy barriers and reaction energies of carbidisation in C-doped Pd structures. As shown in Fig. 3, we considered the relevant transition mechanisms, as demonstrated through the first part of our calculations, from the pristine Pd to pre-formed interstitial carbidic NPs for different C-concentrations per atom fraction (*e.g.*, 1 C atom in Pd_38_ NP corresponds to 2.6% concentration for the PdC system). We observed an increase of the average Pd–Pd bond length with respect to C concentration for all structures as summarized in Table 3. The values obtained through DFT are in agreement with previously published experimental results.^2^

**Table:** The average Pd–Pd bond length for the pristine and C-doped pre-formed structures as calculated through DFT

Pd_38_		Pd_44_		Pd_55_	
C (%)	Pd–Pd (Å)	C (%)	Pd–Pd (Å)	C (%)	Pd–Pd (Å)
0.0	2.74	0.0	2.72	0.0	2.73
2.6	2.75	2.2	2.74	1.8	2.74
9.5	2.79	8.3	2.79	8.3	2.79
15.6	2.83	15.4	2.84	15.4	2.85
20.8	2.88	21.4	2.87	20.3	2.87

**Fig. 4:**
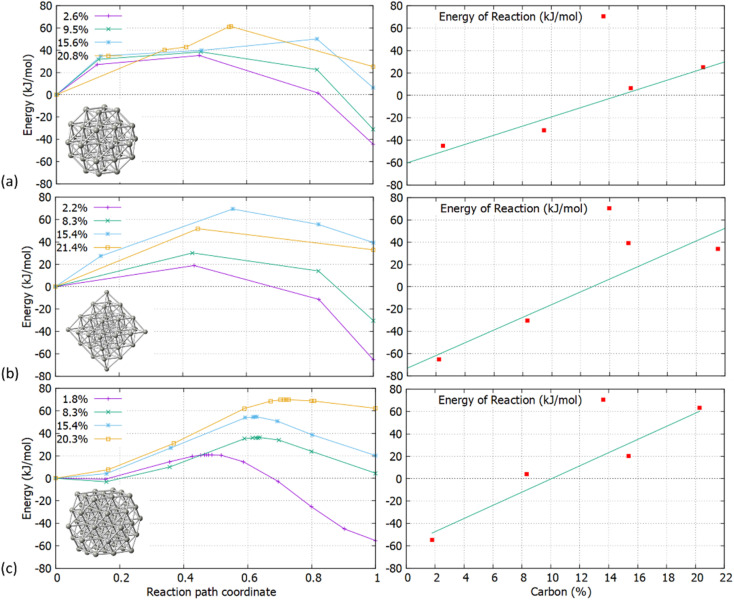
Transition states for the carbidisation processes at increasing C concentrations in (a) Pd_38_ – the left panel shows the activation energies for C concentration at 2.6%, 9.5%, 15.6% and 20.8% and the right panel shows the reaction energies (b) Pd_44_ – the left panel shows the activation energies for C concentration at 2.2%, 8.3%, 15.4% and 21.4% and the right panel shows the reaction energies and (c) Pd_55_ – the left panel shows the activation energies for C concentration at 1.8%, 8.3%, 15.4% and 20.3% and the right panel shows the reaction energies.

The increasing accommodation of C in the Pd lattice was examined *via* the reaction energies between the relevant surface and subsurface configurations. Furthermore, the Pd_6_C formation at approximately 13% of C doping is considered with each Pd atom to be bound with no more than one C atom.^21^ Thermodynamically, it is expected that above a maximum carbidisation threshold, C will reside on the surface rather than migrating into the subsurface region. As shown in Fig. 4, the energy profiles for C incorporation at low concentrations correspond to exothermic reactions. In this case, the final state (products) is more thermodynamically favourable than the initial (reactants), leading to the formation of interstitial Pd carbide. As more C is introduced in the host Pd lattice, the reaction becomes less exothermic and eventually turns to endothermic above a specific concentration for each structure. The maximum C amount that is approximately 14% for Pd_38_ and 11% for Pd_44_/Pd_55_, shows that where the reaction energy becomes positive, further C incorporation is unfavourable and thus C will reside on the surface rather than migrate into the particle.

## 4. Conclusions

In this work, we performed DFT calculations to gain insights on the intrinsic mechanisms of carbidisation in Pd NPs; a process yet not fully understood but important to the catalytic performance of the material in a range of applications. The geometries chosen to represent the Pd NPs structures are the truncated octahedral Pd_38_, octahedral Pd_44_ and cuboctahedral Pd_55_, aiming to account the carbidisation process for both the [100] and [111] facets of the NP structure.

Initially, an individual C atom binds with three surface Pd atoms for the [111] facet and four surface Pd atoms for the [100] facet. In addition, the minimum energy interstitial sites are octahedral where C binds with six Pd atoms in the interior of the particle. Upon insertion of C, minor lattice distortion occurs and the transition results to the occupation of the Pd octahedral interstitial site in the subsurface area, thus our simulations firstly focused on one single C incorporation in the pristine lattice. It is shown that for Pd_38_ and Pd_55_, carbidisation preferentially occurs through the [111] facet where the mechanism corresponds to an exothermic reaction with low activation energy values of 21.2–35.7 kJ mol^−1^. In contrast, for the same mechanism through the [100] facet, an endothermic profile is observed on the reaction with activation energy barriers of more than 120 kJ mol^−1^. Furthermore, the activation energy barriers for interstitial-type diffusion of C through neighbouring sites in the interior of the particle, show preferential residing to the subsurface region (exothermic) rather than closer to the core (endothermic). It is therefore shown that C will migrate from the surface into the octahedral interstitial sites up to relatively limited concentration since it will mainly reside in the subsurface area. Carbidisation at higher concentrations was also investigated using pre-formed carbidic Pd structures to estimate the maximum doping limitations. The distribution of C atoms in the available octahedral sites for low concentrations is within the subsurface area with at least one unoccupied interstitial site between them. Reaction energies for the same mechanism are exothermic up to a 11–14% for all structures whilst beyond this point, further incorporation is energetically unfavourable confirming the limitations of C concentration in Pd NPs.

This study is a first step towards understanding the carbide phase formation that takes place during catalytic reactions with Pd NPs. Future work through experimental and computational studies should further investigate the formation and the role of Pd carbide NPs in heterogeneous catalysis.

## Conflicts of interest

There are no conflicts to declare.

## Supplementary Material

RA-013-D2RA07462A-s001
